# Overreporting of Deaths From Coronary Heart Disease in New York City Hospitals, 2003

**Published:** 2010-04-15

**Authors:** Reena Agarwal, Jennifer M. Norton, Kevin Konty, Regina Zimmerman, Maleeka Glover, Akaki Lekiachvili, Henraya McGruder, Ann Malarcher, Michele Casper, George A. Mensah, Lorna Thorpe

**Affiliations:** Division of General Internal Medicine, Montefiore Medical Center. At the time of the study, Dr Agarwal was affiliated with the New York City Department of Health and Mental Hygiene, New York, New York; New York City Department of Health and Mental Hygiene, New York, New York; New York City Department of Health and Mental Hygiene, New York, New York; New York City Department of Health and Mental Hygiene, New York, New York; Centers for Disease Control and Prevention, Atlanta, Georgia; Centers for Disease Control and Prevention, Atlanta, Georgia; Centers for Disease Control and Prevention, Atlanta, Georgia; Centers for Disease Control and Prevention, Atlanta, Georgia; Centers for Disease Control and Prevention, Atlanta, Georgia; Director, Heart Health and Global Health Policy Global Research and Development PepsiCo 700 Anderson Hill Road, Bldg 6-2 Purchase, NY 10577. At the time of the study, Dr Mensah was affiliated with the Centers for Disease Control and Prevention, Atlanta, Georgia; CUNY School of Public Health at Hunter College, New York, New York. At the time of the study, Dr Thorpe was affiliated with the New York City Department of Health and Mental Hygiene, New York, New York

## Abstract

**Introduction:**

New York City has one of the highest reported death rates from coronary heart disease in the United States. We sought to measure the accuracy of this rate by examining death certificates.

**Methods:**

We conducted a cross-sectional validation study by using a random sample of death certificates that recorded in-hospital deaths in New York City from January through June 2003, stratified by neighborhoods with low, medium, and high coronary heart disease death rates. We abstracted data from hospital records, and an independent, blinded medical team reviewed these data to validate cause of death. We computed a comparability ratio (coronary heart disease deaths recorded on death certificates divided by validated coronary heart disease deaths) to quantify agreement between death certificate determination and clinical judgment.

**Results:**

Of 491 sampled death certificates for in-hospital deaths, medical charts were abstracted and reviewed by the expert panel for 444 (90%). The comparability ratio for coronary heart disease deaths among decedents aged 35 to 74 years was 1.51, indicating that death certificates overestimated coronary heart disease deaths in this age group by 51%. The comparability ratio increased with age to 1.94 for decedents aged 75 to 84 years and to 2.37 for decedents aged 85 years or older.

**Conclusion:**

Coronary heart disease appears to be substantially overreported as a cause of death in New York City among in-hospital deaths.

## Introduction

Coronary heart disease (CHD) is the leading cause of death for adults in the United States, and stroke ranks third (1). In New York City, an unusual pattern of high CHD death rates and low stroke death rates has been observed; the age-adjusted CHD death rate in 2003 was 1.7 times the national rate, and the age-adjusted stroke death rate was half the national rate (1,2). This pattern is unexpected, given that risk factors for CHD and stroke are similar and that the prevalence in New York City of most common CHD risk factors, such as hypertension, hyperlipidemia, obesity, and smoking, is similar to or lower than that in the rest of the country (3). New York City CHD death rates have been consistently higher than national CHD death rates for more than 3 decades despite steady CHD death rate declines nationally and in New York City (Figure).

**Figure 1.**
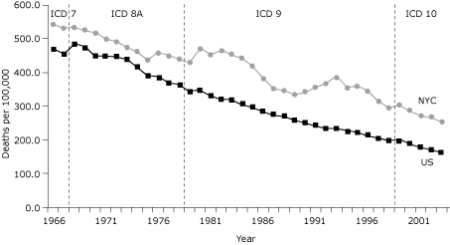
Age-adjusted deaths from coronary heart disease in New York City (NYC) versus the United States overall. "ICD" indicates the revisions of the International Classification of Diseases that were used to categorize cause of death. US data for 1950 through 2002 from National Heart, Lung, and Blood Institute, 2002. US data for 2003 through 2006 from Centers for Disease Control and Prevention. NYC data from NYC Department of Health and Mental Hygiene, Bureau of Vital Statistics. NYC population data from 1971 through 2003 census estimates. NYC population data from 1961 through 1969 from linear interpolation.

**Table:** 

**ICD/Year**	**United States**	**New York City**
**ICD-7**		
1966	465.1	538.4
1967	453.9	530.0
**ICD-8A**		
1968	482.6	529.9
1969	469.8	521.4
1970	448.0	512.6
1971	448.5	496.7
1972	445.5	489.2
1973	437.0	471.1
1974	414.6	461.7
1975	388.1	435.4
1976	382.2	452.7
1977	368.5	449.2
1978	362.0	436.0
**ICD-9**		
1979	340.5	427.8
1980	345.2	467.8
1981	329.5	451.5
1982	320.3	460.1
1983	316.0	450.9
1984	304.1	441.3
1985	296.2	417.4
1986	283.3	378.3
1987	273.9	351.5
1988	268.5	345.8
1989	257.5	333.7
1990	249.6	341.5
1991	240.6	354.6
1992	232.6	366.0
1993	233.2	383.2
1994	224.5	352.3
1995	219.7	355.4
1996	212.1	340.8
1997	203.6	313.4
1998	197.1	292.9
**ICD-10**		
1999	195.7	302.9
2000	186.6	285.7
2001	177.8	269.3
2002	170.6	266.5
2003	162.8	253.9

Misreporting cause of death on death certificates may be contributing to New York City's observed high CHD death rate. Studies have suggested that such misreporting may be common. A study of 2 Texas military hospitals found that 37% of death certificates reported a different cause of death than did autopsy (4). A Swedish study found that 54% of death certificates reported a different cause of death than did chart review (5). The level of misreporting varies by cause of death. For CHD, a British study found that death certificates have a low sensitivity for CHD deaths compared with autopsy findings (6). A study of Framingham Heart Study participants found that death certificates attributed 24% more deaths to CHD than did a physician panel that reviewed medical records (7). The Atherosclerosis Risk in Communities (ARIC) study also found that death certificates overestimated CHD deaths by 20% compared with a physician review panel (8).

We sought to determine whether local physician reporting patterns of CHD on death certificates contribute to the observed pattern of high-CHD, low-stroke death rates observed in New York City. Our investigation was designed to determine the accuracy of death certificate reporting of CHD as a cause of death in New York City by using methods employed by the ARIC study in other jurisdictions. We did not directly address accuracy of death certificate reporting of stroke in this analysis.

## Methods

### Study design and population

We developed the study design by using the methods of the ARIC study, which evaluated CHD reporting on death certificates in 4 geographically distinct US communities by using a blinded medical record review process (8). A completed “determination of research status” form was submitted to the Centers for Disease Control and Prevention (CDC), which determined that this study would not require institutional review board approval. We assessed New York City death certificates dated January through June 2003, including those for which the decedent 1) was a resident of New York City, 2) died in one of the city’s 70 hospitals, and 3) had an underlying cause of death on his or her death certificate that corresponded to a subset of codes associated with CHD or stroke from the International Classification of Diseases, 10th revision (ICD-10) and corresponding to the ICD-9 codes used in the ARIC validation study (Table 1). A data set with 13,144 eligible death certificates, including 7,674 for in-hospital deaths, was obtained from the New York City Department of Health and Mental Hygiene (DOHMH), Bureau of Vital Statistics. We took a stratified random sample based on New York City neighborhoods with low, medium, or high age-adjusted CHD death rates in 2001; each stratum contained roughly one-third of the sampled population. This study was restricted to 491 in-hospital deaths to maximize feasibility of data collection. In-hospital deaths were categorized as inpatient, emergency department (ED), outpatient, and dead on arrival (DOA).

### Data collection

Hospitals provided medical records for the sampled decedents. We abstracted information from each record and entered it into a standardized computer-based abstraction form developed by using EpiInfo version 3.4.1 (CDC, Atlanta, Georgia). This form contained pre-populated fields for demographic data reported on the death certificates. Information abstracted from the medical chart included time from onset of symptoms to death, presence of chest pain, history of CHD, and medication use. We photocopied electrocardiogram results (ECGs), test results for cardiac enzymes, and medical discharge summaries.

### External review

Two external teams were assembled, each consisting of 2 physician epidemiologists, trained and certified in the ARIC protocol for validation of CHD events. They were blinded to the cause of death listed on the death certificate and used case summary reports generated from the medical record abstraction, ECGs, and medical discharge summaries to answer 5 yes/no questions about each case: 1) Was there a known nonatherosclerotic or noncardiac process or event that was probably lethal?, 2) Was there a definite myocardial infarction (MI) for which the patient was hospitalized within 4 weeks of death?, 3) Was there a probable MI for which the patient was hospitalized within 4 weeks of death?, 4) Was there a history of chest pain within 72 hours of death?, and 5) Was there a history of ever having had chronic ischemic heart disease such as coronary insufficiency or angina? When initial responses to questions differed within each team, the 2 reviewers discussed the case until they agreed on a final determination.

### Case definitions

Death certificate CHD deaths were defined as ICD-10 codes I20-I25 and I51.6. Death certificate non-CHD deaths were defined as all other ICD-10 codes included in the study (Table 1). We used an algorithm based on the ARIC methods, incorporating the reviewer responses to the 5 yes/no questions described above, to develop 2 definitions of CHD: definite CHD and possible CHD. Definite CHD was defined as 1) no known nonatherosclerotic or noncardiac process or event that was probably lethal and 2) at least 1 of the following conditions: definite MI for which the patient was hospitalized within 4 weeks of death, history of chest pain within 72 hours of death, or history of chronic ischemic heart disease such as coronary insufficiency or angina pectoris. The definite CHD validation definition relies heavily on information available in the medical record. To account for the possibility that a death may be due to CHD even if relevant information is not clearly documented in the chart, we used a second case definition, possible CHD. Possible CHD was defined as 1) no known nonatherosclerotic or noncardiac process or event that was probably lethal and 2) a death certificate with a consistent underlying cause of death (ICD-10 codes I20-I25 and I51.6). This definition is more sensitive, excluding only those deaths with a clearly recorded cause other than CHD.

### Data analysis

The goal of data analysis was to estimate the degree to which CHD deaths were reported on death certificates when measured against the standard of external medical chart review. To obtain this measurement, we calculated comparability ratios (CRs) as the number of CHD deaths defined by the death certificate divided by the number of CHD deaths defined by review of the medical record. We also calculated sensitivity, false-positive rate (FPR), and positive predictive value (PPV).

The final data set contained records that were abstracted and validated (n = 444). Observations were assigned initial weights equal to their inverse probability of selection, which varied by strata. The target population consisted of all New York City in-hospital decedents with sampled ICD-10 codes (n = 7,800) who died during the study period. We used post-stratification weighting to account for differences in race/ethnicity and sex distributions between the sample and the target population. To account for the complex sampling design, we computed variance estimates for CRs by using a jackknife estimator for stratified samples (9).

For direct comparison to the ARIC study of inpatient deaths (excluding ED, outpatient, and DOA deaths), we also calculated survey weights and outcome measures for the inpatient subpopulation. Findings were similar for the inpatient-only and comprehensive samples; thus, detailed results are presented only for the larger in-hospital sample.

## Results

Of the 491 eligible cases, 444 records (90%) were abstracted from hospital charts and assessed by the reviewers. Charts were unavailable for the remaining 47 cases. Of the 444 decedents, 345 were inpatients; 70 were ED patients or outpatients; 23 were DOA, and 6 died in other or unknown places in the hospital (Table 2). Most were women (54%), aged 75 years or older (66%), non-Hispanic white (55%), and died in a private hospital (89%).

Reviewer disagreement before adjudication on each of the 5 validation questions ranged from 5% to 31%. The sensitivity of death certificates for definite CHD deaths was 0.87, the FPR was 0.66, and the PPV was 0.46 (Table 3). The overall CR for definite CHD was 1.91 (95% confidence interval [CI], 1.59-2.23). The CR increased with age, from 1.51 for decedents aged 35 to 74 years to 2.37 for decedents aged 85 years or older. The CR was 1.83 for women and 2.01 for men; it was 2.08 for whites, 2.14 for blacks, and 1.30 for Hispanics. When the broader possible CHD definition was used, the sensitivity was 0.91, the PPV was 0.66, and the FPR was 0.54. The CR for possible CHD deaths was 1.37 (95% CI, 1.21-1.53).

When data were stratified by neighborhoods with low, medium, and high CHD death rates, the CR increased progressively from low to high strata. The CR for definite CHD in the low stratum was 1.82 (95% CI, 1.40-2.24), in the medium stratum was 2.06 (95% CI, 1.67-2.45), and in the high stratum was 2.79 (95% CI, 2.43-3.15). This trend remained consistent in the inpatient population (CR range, 1.71-2.80) and when using the "possible CHD" validation definition for both the total in-hospital population (CR, 1.28-1.99) and the inpatient-only population (CR, 1.30-2.36).

## Discussion

These results demonstrate substantial overreporting of CHD as a cause of death on death certificates in New York City for in-hospital deaths, when measured against the standard of medical record review. The CR for definite CHD was 1.51 for in-hospital decedents and 1.33 for inpatient decedents (in-hospital deaths excluding DOA, outpatient, and ED deaths) aged 35 to 74 years. The inpatient population is comparable to the inpatient population in the ARIC study, which examined deaths among decedents aged 35 to 74 years in 4 other sites across the country. The ARIC study found modest underreporting of CHD deaths on death certificates compared with chart review by using the same validation definition for definite CHD (CR, 0.9). These results indicate that overreporting of CHD on death certificates may contribute to the elevated CHD death rates observed in New York City.

The study design was based on the methods of the ARIC validation study but differs from the ARIC study in 3 ways. First, the ARIC study was conducted from 1987 through 1995, when ICD-9 was used to code cause of death, whereas this study was conducted in 2003 by using ICD-10 codes. Given the high correspondence between ICD-9 and ICD-10 for CHD (10), the change in classification system is unlikely to account for the differences in the CRs between ARIC and this study. Second, stroke deaths reported on death certificates were not included in ARIC but were included in our study. Including stroke deaths could have resulted in a greater opportunity for false or true negatives. False negatives would have resulted in a lower CR. However, our sample included only a small number of stroke deaths, and all were classified as true negatives. Therefore, the inclusion of stroke deaths did not affect our comparison with ARIC. Third, this study included a high proportion of decedents aged 75 years or older, many of whom had multiple chronic medical problems; the presence of these comorbidities made validation especially difficult, as evidenced by the range of initial disagreement between the reviewers on the 5 validation questions. Difficulty in validating cause of death was also due to conflicting, sparse, or missing information in hospital charts. When reviewers were uncertain how to answer a question, they chose "no," frequently resulting in a validated "No CHD" classification. This in turn yielded fewer validated CHD deaths, possibly inflating the CR.

To our knowledge, this is the first study to examine CHD reporting on in-hospital death certificates in New York City. The findings have implications for public health and vital registration practice. Further work is needed to better understand patterns of death certificate completion for in-hospital deaths. Providers who are most knowledgeable about the patient do not always complete the death certificate; that task may be assigned to residents or fellows who are not properly instructed in completion, leading to inaccurate reporting (11). In addition, providers who complete the death certificate may not have all relevant patient information available at that time. In particular, physicians in the ED have limited information at the time of death certificate completion. External reviewers may have had the benefit of pathology or laboratory reports unavailable to the provider at the time of death certificate completion. Lack of information about DOA, outpatient, and ED decedents may account for the difference between inpatient and in-hospital CRs.

Other reasons for CHD overreporting on death certificates may be more specific to New York City. New York City has a central registration process, but the rest of the state has approximately 1,500 local registrars, similar to most other health jurisdictions. Therefore, any bias in reporting, such as misunderstanding regarding how death certificates should be completed, may be compounded. For example, in the past the New York City burial desk rejected death certificates for improper completion of cause of death. This rejection process no longer occurs, but the fear of having a death certificate rejected may still lead many physicians to complete certificates with common and "acceptable" causes of death, such as CHD. Second, New York City requires death certificates to be registered within 72 hours of death. This time pressure may result in more frequent reporting of certain causes of death that are easier to assume in a decedent with many comorbidities, such as older decedents. Third, the large number of teaching hospitals in New York City may compound the problem of providers who are less familiar with the decedent's medical history being responsible for completing death certificates.

This study highlights the possibility that New York City's observed high rate of CHD may be partly due to misreporting of CHD on death certificates. Death certificate accuracy may be improved by physician education. The city's Bureau of Vital Statistics has developed an online tool to educate providers in all phases of their careers about death certificate completion, and other education materials are being developed (12). As the success of these efforts are evaluated, it will be important to monitor trends in the reporting of CHD deaths on death certificates and to assess any changes in the CHD death rates that could be due to changes in reporting practices. Meanwhile, CHD is the leading cause of death in New York City, and efforts to reduce its burden remain a top public health priority. Having accurate data will help DOHMH meet its mission to protect and promote the health of all New Yorkers.

## Acknowledgments

Data collection for this study was partially supported by the Centers for Disease Control and Prevention, Epidemiology Program Office.

## Figures and Tables

**Table 1:** ICD-9 and ICD-10 Codes Used to Report CHD as a Cause of Death on Death Certificates, New York City, 2003

**Description of ICD-9 Code**	ICD-9	ICD-10	**Description of ICD-10 Code**
**Included in ARIC study^a^**			
Diabetes mellitus	250	E10	Insulin-dependent diabetes mellitus
Diabetes mellitus	250	E11	Non–insulin-dependent diabetes mellitus
Diabetes mellitus	250	E12	Malnutrition-related diabetes mellitus
Diabetes mellitus	250	E13	Other specified diabetes mellitus
Diabetes mellitus	250	E14	Unspecified diabetes mellitus
Essential hypertension	401	I10	Essential hypertension
Hypertensive heart disease	402	I11	Hypertensive heart disease
Angina pectoris	413	I20	Angina pectoris
Acute MI	410	I21	Acute MI
Acute MI	410	I22	Subsequent MI
—b	—b	I23	Certain current complications following acute MI
Other acute and subacute IHD	411	I24	Other acute IHD
Old MI, other forms of chronic IHD	412, 414, 429.2	I25	Chronic IHD
Cardiac dysrhythmias	427	I46-I49	Cardiac arrest; paroxysmal tachycardia; atrial fibrillation; other cardiac arrhythmias
Heart failure	428	I50	Heart failure
Ill-defined descriptions and complications of heart disease	429 but not 429.2	I51	Ill-defined complications of heart disease
Atherosclerosis	440	I70	Atherosclerosis
Acute edema of lung, unspecified	518.4	J81	Pulmonary edema
Sudden death, cause unknown	798	R96	Other sudden death, cause unknown
Other ill-defined and unknown	799	R99	Other ill-defined and unknown cause
**Not included in ARIC study**			
Subarachnoid hemorrhage	430	I60	Subarachnoid hemorrhage
Intracerebral hemorrhage	431	I61	Intracerebral hemorrhage
Other and unspecified intracranial hemorrhage	432	I62	Other nontraumatic intracranial hemorrhage
Cerebral artery occlusion with infarction	434.9	I63	Cerebral infarction
Acute but ill-defined cerebrovascular disease	436	I64	Stroke, not specified as hemorrhage or infarction
Occlusion and stenosis of precerebral arteries	433	I65	Occlusion and stenosis of precerebral arteries
Occlusion of cerebral arteries	434	I66	Occlusion of cerebral arteries
Other and ill-defined cerebrovascular disease	437	I67	Other cerebrovascular diseases
Other and ill-defined cerebrovascular disease	437	I68	Cerebrovascular disease in diseases classified elsewhere
Late effects of cerebrovascular disease	438	I69	Sequelae of cerebrovascular disease

Abbreviations: ICD-9, International Classification of Diseases, 9th revision; ICD-10, International Classification of Diseases, 10th revision; CHD, coronary heart disease; ARIC, Atherosclerosis Risk in Communities; MI, myocardial infarction; IHD, ischemic heart disease.

a The ARIC study evaluated coronary heart disease reporting on death certificates in 4 geographically distinct US communities using a blinded medical record review process (8).

a No analogous code.

**Table 2:** Characteristics of Decedents for Whom Cause of Death Was Reported as CHD on Death Certificates, New York City, 2003

**Characteristic**	No. (n = 444)	Weighted No. (n = 7,800)	Weighted %^a^
**Demographic Characteristics**			
**Sex**			
Women	249	4,190	54
Men	195	3,610	46
**Age, y**			
<35	2	15	<1
35-74	143	2,664	34
≥75	299	5,121	66
**Race/ethnicity**			
Non-Hispanic white	276	4,262	55
Non-Hispanic black	103	1,814	23
Hispanic	34	1,038	13
Other/missing/unknown	31	686	9
**Mortality Characteristics**			
**Hospital type**			
Private	406	6,927	89
Public	38	873	11
**Place of death**			
Inpatient	345	5,923	76
Outpatient/ED/DOA	93	1,776	23
Other place	5	86	1
Unknown	1	15	<1
**Death certificate cause of death^b^**			
ICD-10: I20-I25	365	5,773	74
ICD-10: I21 only	63	1,109	14
ICD-10: I25 only	300	4,627	59
Other (not I20-I25)	79	2,027	26

Abbreviations: CHD, coronary heart disease; ED, emergency department; DOA, dead on arrival; ICD-10, International Classification of Diseases, 10th revision.

a Percentages may not total 100 because of rounding.

b More than 1 ICD code may be recorded per record.

**Table 3:** Validation Measures of Death Certificate Accuracy for Reporting CHD, New York City, 2003

**Characteristic**	Sensitivity^a^	Positive Predictive Value^b^	False-Positive Rate^c^	Comparability Ratio^d^ (95% CI)
**Definite CHD^e^**				
**Age, y**				
35-74	0.77	0.51	0.53	1.51 (1.07-1.95)
75-84	0.88	0.46	0.69	1.94 (1.33-2.55)
≥85	0.98	0.41	0.75	2.37 (1.71-3.02)
**Sex**				
Women	0.88	0.48	0.63	1.83 (1.46-2.20)
Men	0.86	0.43	0.69	2.01 (1.45-2.57)
**Race/ethnicity**				
Non-Hispanic white	0.92	0.44	0.72	2.08 (1.66-2.50)
Non-Hispanic black	0.83	0.39	0.59	2.14 (1.21-3.06)
Hispanic	0.83	0.64	0.37	1.30 (0.79-1.81)
**Total in-hospital**	0.87	0.46	0.66	1.91 (1.59-2.23)
**Total inpatient^f^**	0.82	0.44	0.66	1.89 (1.49-2.29)
**Possible CHD^g^**				
**Age, y**				
35-74	0.84	0.78	0.34	1.07 (0.89-1.26)
75-84	0.91	0.62	0.61	1.48 (1.11-1.85)
≥85	0.99	0.61	0.67	1.62 (1.33-1.91)
**Sex**				
Women	0.92	0.69	0.50	1.32 (1.14-1.50)
Men	0.90	0.62	0.59	1.44 (1.15-1.73)
**Race/ethnicity**				
Non-Hispanic white	0.94	0.61	0.64	1.54 (1.32-1.76)
Non-Hispanic black	0.90	0.72	0.40	1.26 (0.96-1.56)
Hispanic	0.87	0.90	0.13	0.96 (0.75-1.17)
**Total in-hospital**	0.91	0.66	0.54	1.37 (1.21-1.53)
**Total inpatient^f^**	0.86	0.59	0.59	1.46 (1.22-1.70)

Abbreviation: CHD, coronary heart disease; CI, confidence interval.

a CHD defined by death certificate and by validation divided by CHD defined by validation.

b CHD defined by death certificate and by validation divided by CHD defined by death certificate.

c CHD defined by death certificate but not by validation divided by all death certificates validated as not CHD.

d CHD defined by death certificate divided by CHD deaths defined by validation.

e Defined as 1) no known nonatherosclerotic or noncardiac process or event that was probably lethal and 2) at least 1 of the following conditions: definite myocardial infarction for which the patient was hospitalized within 4 weeks of death, history of chest pain within 72 hours of death, or history of chronic ischemic heart disease such as coronary insufficiency or angina pectoris.

f Inpatient sample is equivalent to the in-hospital sample excluding outpatient, emergency department, and dead on arrival deaths.

g Defined as 1) no known nonatherosclerotic or noncardiac process or event that was probably lethal and 2) a death certificate with a consistent underlying cause of death.
